# Incorporating functional inter-relationships into protein function prediction algorithms

**DOI:** 10.1186/1471-2105-10-142

**Published:** 2009-05-12

**Authors:** Gaurav Pandey, Chad L Myers, Vipin Kumar

**Affiliations:** 1 Department of Computer Science & Engineering, University of Minnesota, Minneapolis, MN, USA

## Abstract

**Background:**

Functional classification schemes (e.g. the Gene Ontology) that serve as the basis for annotation efforts in several organisms are often the source of gold standard information for computational efforts at supervised protein function prediction. While successful function prediction algorithms have been developed, few previous efforts have utilized more than the protein-to-functional class label information provided by such knowledge bases. For instance, the Gene Ontology not only captures protein annotations to a set of functional classes, but it also arranges these classes in a DAG-based hierarchy that captures rich inter-relationships between different classes. These inter-relationships present both opportunities, such as the potential for additional training examples for small classes from larger related classes, and challenges, such as a harder to learn distinction between similar GO terms, for standard classification-based approaches.

**Results:**

We propose a method to enhance the performance of classification-based protein function prediction algorithms by addressing the issue of using these interrelationships between functional classes constituting functional classification schemes. Using a standard measure for evaluating the semantic similarity between nodes in an ontology, we quantify and incorporate these inter-relationships into the *k*-nearest neighbor classifier. We present experiments on several large genomic data sets, each of which is used for the modeling and prediction of over hundred classes from the GO Biological Process ontology. The results show that this incorporation produces more accurate predictions for a large number of the functional classes considered, and also that the classes benefitted most by this approach are those containing the fewest members. In addition, we show how our proposed framework can be used for integrating information from the entire GO hierarchy for improving the accuracy of predictions made over a set of base classes. Finally, we provide qualitative and quantitative evidence that this incorporation of functional inter-relationships enables the discovery of interesting biology in the form of novel functional annotations for several yeast proteins, such as Sna4, Rtn1 and Lin1.

**Conclusion:**

We implemented and evaluated a methodology for incorporating interrelationships between functional classes into a standard classification-based protein function prediction algorithm. Our results show that this incorporation can help improve the accuracy of such algorithms, and help uncover novel biology in the form of previously unknown functional annotations. The complete source code, a sample data set and the additional files for this paper are available free of charge for non-commercial use at .

## Background

A variety of recently available high throughput data sets, such as protein-protein interaction networks, microarray data and genome sequences, offer important insights into the mechanisms leading to the accomplishment of a protein's function. However, the complexity of analyzing these data sets manually has motivated the development of numerous computational approaches for predicting protein function [1,2]. For a recent comprehensive survey on this topic, see Pandey *et al *(2006) [3].

One of the most popular methods used for predicting protein function from biological data is classification [4-6]. Under the traditional classification framework, each protein is represented by a set of features, such as the expression profile of its corresponding gene or the set of proteins it interacts with. Now, for each functional class, a model is constructed using the feature sets of the proteins annotated with this class. This model is then used to decide if an unannotated query protein should be annotated with this class. The key premise underlying this methodology for predicting protein function is that proteins belonging to the same functional class have "similar" biological attributes.

Standard classification or predictive modeling techniques for function prediction rely on positive and negative examples from functional classification schemes, such as the Gene Ontology [7] or FunCat [8], and typically treat each functional class separately. However, this standard approach fails to capture one of the key properties of such classification schemes: most schemes not only provide annotations to functional classes, but also capture inter-relations between the functional classes. For example, the Gene Ontology (GO) is arranged as a directed acyclic graph in which the GO terms form a hierarchy capturing everything from relatively general functions (e.g. metabolism) to specific biological roles (e.g. nucleotide excision repair). Such an organization of classes, particularly in the case of GO, poses two important challenges for predictive modeling techniques for function prediction. First, several studies [9-11] have concluded that proteins in inter-related, or *similar *GO functional classes tend to have similar biological attributes. This limits the applicability of the key premise of classification-based function prediction discussed above, since distinguishing between such similar GO classes becomes hard.

**Figure 1:**
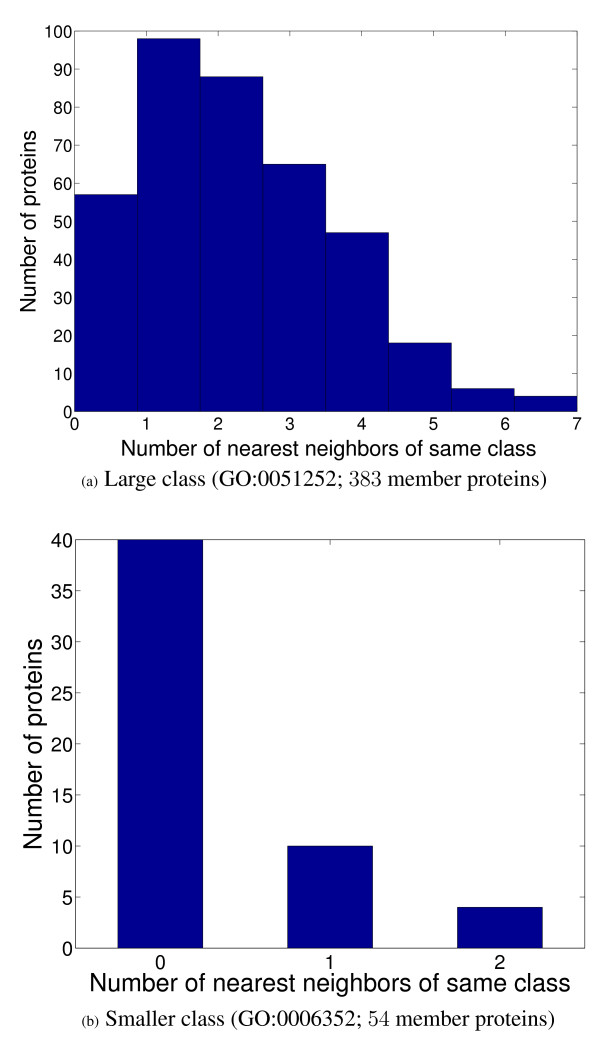
**Distribution of the number of nearest neighbors of the same class in a neighborhood of size 20 for the member proteins of two functional classes of varying sizes in Mnaimneh *et al*'s data set**.

The second important issue that arises is that it is often hard to construct reliable classification models for several functional classes from a given data set due to complex issues including noise in the data, low relevance of the data set for some functional classes, and an insufficient number of training examples for building accurate classification models [12]. These issues, particularly the last one, are expected to most severely affect functional classes having few members, which also include classes located deep in a functional hierarchy. This difficulty of constructing reliable classification models is illustrated in Figure 1 for two classes from the GO biological process ontology, which have 383 and 54 member proteins in Mnaimneh *et al*'s gene expression data set [13], representing a large class (GO:0051252) and a class of median size (GO:0006352) respectively. The histograms in this figure show, for each protein in these classes, how many proteins in its neighborhood belong to the same class. Neighborhood is restricted to the twenty proteins with the most similar expression profiles to the query protein, using correlation as the similarity measure. These plots show that for most of the proteins in both the large, as well as the much smaller class, only a limited number of similar proteins in the same class are available. For instance, in the large class, 243 of the 383 member proteins have less than three similar proteins in the same class, while two is the maximum number of neighboring proteins of the same class for proteins in the smaller class. In fact, 40 of the 54 proteins in the smaller class have no proteins of the same class in their neighborhood. This lack of enough training examples having characteristics similar to the query protein, which occurs due to the issues discussed above, illustrates the difficulty of building classification models for functional classes, particularly for the small ones.

**Figure 2:**
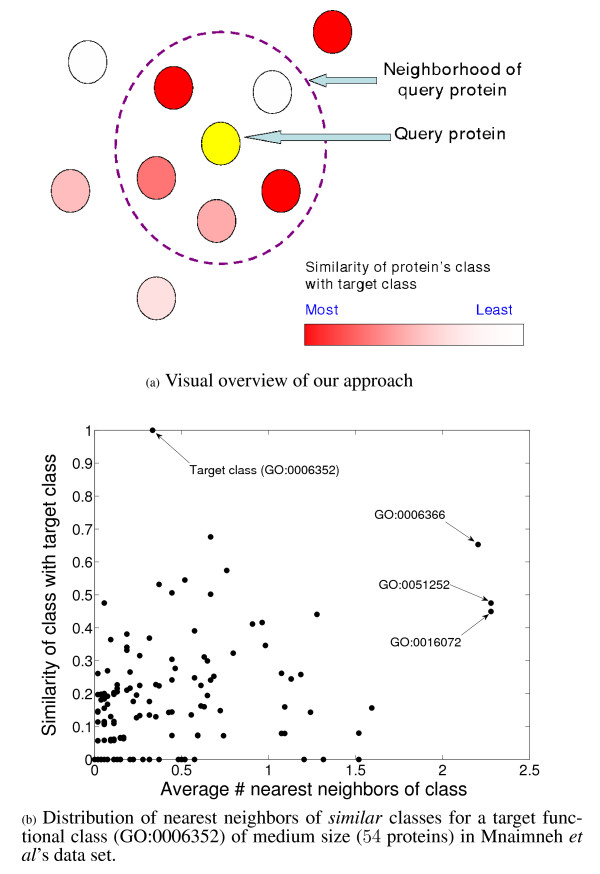
**Conceptual visualization of our approach for incorporating functional inter-relationships into function predictions algorithms, and empirical evidence to show the abundance of proteins carrying labels *similar *to the target class in the neighborhood of a query protein **(a) Overview of our approach. In addition to using the proteins annotated with the target class (dark red circles) in the query protein's neighborhood, our approach also uses evidence from proteins annotated with a class *similar *to the target class (lighter red circles) in order to determine whether the query protein belongs to the target class (b) Distribution of nearest neighbors of *similar *classes for a target functional class (GO:0006352) of medium size (54 proteins) in Mnaimneh *et al*'s data set. The Y-axis denotes the semantic similarity of a functional class with the target class, and the X-axis shows the average number of proteins in that class that are included in a size 20 neighborhood of proteins annotated with the target class. Some of the classes (marked by arrows) have a significant similarity with the target class, and have an average frequency higher than that of the target class (similarity = 1) itself.

However, the availability of the same well-defined structure of relationships between functional classes in the form of Gene Ontology raises the following key question: "Can the performance of standard classification algorithms for function prediction be improved by incorporating these inter-relationships into them?". In this paper, we address this question using an approach shown visually in Figure 2(a). As illustrated by this figure, our approach uses evidence in neighboring proteins belonging to similar classes to bolster the evidence for annotation of the query protein with the target class. Evidence for the abundance of proteins belonging to classes similar to the target class in the neighborhood of a query protein, and hence the applicability of such an approach is presented in Figure 2(b) for the target class of median size (GO:0006352) discussed in Figure 1(b). Here, the semantic similarity of all the classes with the target class, calculated using Lin's measure [14], is plotted against the average number of nearest neighbors of the corresponding class in the nearest neighborhood of a protein belonging to the target class. As can be seen from this scatter plot, even though the average frequency of the target class (similarity = 1) is very small (less than 0.5), there are several classes, such as GO:0006366, GO:0051252 and GO:0016072, that are more abundant, and have a substantial semantic similarity with the target class (over 0.4). This similarity can be used to enrich the information available in the neighborhood of proteins being tested for a target class. Our approach is based on this principle.

More specifically, using Lin's measure [14] for evaluating the semantic similarity between classes in an ontology, we incorporate such functional interrelationships into the *k*-nearest neighbor classifier [4]. We evaluate our algorithm on two large microarray data sets [13,15], a recent protein interaction data set [16] and a combination of interaction and microarray data sets, each of which is used for the modeling and prediction of over hundred classes from the GO Biological Process ontology. The results show that, compared to the base k-NN classifier, this incorporation produces more accurate predictions for many of the functional classes considered, and also that the classes benefitted most by this approach are those containing the fewest members. We also illustrate how the proposed framework can be used for integrating information in the entire GO Biological Process ontology to improve the accuracy of prediction over a set of target classes. Finally, we provide qualitative and quantitative evidence that this incorporation of functional inter-relationships enables the discovery of interesting biology in the form of novel functional annotations for several yeast proteins, such as Sna4, Rtn1 and Lin1.

Note that since the rest of the discussion in this paper will be concerned with classification within the context of GO, the terms (functional) *class*, (GO) *term*, *node *(in an ontology) and *label *will be used interchangeably in the rest of the text.

## Related Work

Recently, some approaches have been proposed to address the problem of incorporating inter-relationships between functional classes in GO into function prediction algorithms. These approaches can be categorized using the following two types of relationships between classes constituting the DAG-based functional hierarchies in GO:

### Parent-child relationships

The basic structure of the ontologies in GO is constructed from edges between parent and children terms. Some approaches have recently been proposed for enforcing the consistency required by these relationships, namely a protein annotated with a child node must be annotated with the parent node, into function prediction algorithms. Barutcuoglu *et al *[17] proposed a Bayesian network-based approach for this incorporation. In this work, they trained individual SVM classifiers on all the nodes of the hierarchy. Then, by constructing a Bayesian network using the structure of the ontology, the predictions of all the nodes were corrected iteratively in order to ensure consistency between parent-child annotations throughout the hierarchy, obtaining significant improvements over the individual classifiers. Carroll and Pavlovic [18] proposed a similar approach using probabilistic chain graphs for this problem. However, due to the limited evaluation experiments on small hierarchies, it is unclear how the performance of this approach would scale for a large set of classes from GO. Some other researchers, such as Shahbaba and Neal (2006) [19], have also studied this problem, although their techniques are limited to tree-structured hierarchies.

### Sibling and other distant relationships

An effect of the structure of the ontologies in GO is the formation of sibling relationships between nodes that are children of the same parent. These relationships can be further generalized to extended family relationships, such as cousin and other more distant relationships. King *et al *[20] approached the problem of incorporating these distant relationships into function prediction algorithms by predicting the functions of a protein using the decision tree and Bayesian network models trained on the patterns of annotations of other proteins. Tao *et al *[21] extended King *et al*'s approach further by augmenting the prediction model with the semantic similarity between different classes. Here, they used Lin's similarity measure [14], also used in our study but using a different definition, to measure the interrelationships between the functional classes in GO, and thus to measure the similarity between the sets of functional labels of two proteins. This similarity measure is then used within the framework of a k-nearest neighbor classifier for predicting whether an unannotated protein belongs to a certain functional class or not. The results of this study provided important evidence for the utility of semantic similarity between functional classes for enhancing the performance of function prediction algorithms. However, since this technique uses the known annotations of a protein to predict its other potential annotations, it can not make predictions for proteins with no known annotations, since such a protein will have no similarity to the other proteins using this measure. This motivates the need for methods that can incorporate external genomic data into the prediction process, so that similarity can be computed for both characterized and previously unannotated proteins alike. Our work takes this approach of augmenting biological data-based functional classification algorithms with inter-relationships between functional classes measured using standard semantic similarity measures. Notably, Yu *et al *have recently proposed a similar approach for this problem [22], where they use taxonomic similarity measures between functional classes to modify a simple protein function prediction algorithm. We compare our method with this approach.

The incorporation of both these types of relationships is important for making use of the information available in the entire hierarchy. One of the advantages of the direct incorporation of distant functional relationships, which is the focus of the latter set of studies, is that it is possible to incorporate information from nodes farther away in the hierarchy, as compared to the hierarchical incorporation approaches, which only utilize the subgraph of the hierarchy corresponding to the set of target classes. Our work provides a framework for incorporating these distant functional inter-relationships into standard function prediction algorithms. Notably, this task is more challenging than the hierarchical consistency enforcement problem since there is a much larger number of relationships between nodes to be considered than just the parent-child relationships, which are relatively fewer. This factor makes the incorporation of non-hierarchical relationships more challenging. Furthermore, as discussed above, we perform this augmentation using the biological characteristics of proteins captured in high-throughput genomic data, thus addressing one of the limitations of King *et al*'s [20] and Tao *et al*'s [21] studies. This enables us to make predictions for poorly annotated and unannotated proteins, for which experimental data, such as their interactions and expression profiles, are available. Our experimental results provide both qualitative and quantitative evidence for this advantage of our approach. We also present the results of a comparison between the performance of our and Yu *et al*'s GEST [22] approach.

Our work is also related to the field of hierarchical and multi-label classification in machine learning and data mining [23]. However, most of the work in this field is not directly applicable to the problem of hierarchy-based protein function prediction, since these techniques don't take the hierarchical and multi-label nature of this classification simultaneously into account. Also, they consider limited, if any, relationships between the classes, which is the primary subject of this study.

## Methods

### Preliminaries

#### Semantic Similarity in an Ontology

In GO, nodes (classes or labels) are connected to other nodes through parent-child edges, which impose hierarchical inter-relationships between them. Also, the nodes contain member proteins that have been annotated with the corresponding functional class. Thus, it is possible to compute the similarity between two GO nodes, referred to as *semantic similarity*, on the basis of their relative positioning in the hierarchy, their contents, or a combination of both. Several information-theoretic semantic similarity measures have been developed for computing similarity between two concepts in a hierarchy, such as those by Lin [14], Resnik [24] and Jiang [25]. These measures evaluate the similarity of two nodes in terms of their proximity in the ontology, as well as their content. In particular, we use Lin's measure [14] as defined in Equation 1.

(1)

Here, *l*_1 _and *l*_2 _are the labels (or nodes) between which similarity is being calculated, while *p*(*l*) denotes the probability of a protein being annotated with label *l*, and is estimated from the available set of GO annotations for an organism. Also, , where *S*(*l*_1_, *l*_2_) is the set of common ancestors of *l*_1 _and *l*_2_. Thus, *p*_*ms*_(*l*_1_, *l*_2_) denotes the probability of the *minimum subsumer *of *l*_1 _and *l*_2_. Intuitively, this formulation measures the semantic similarity of *l*_1 _and *l*_2 _in terms of the contents of their minimum subsumer node in the ontology. Clearly, *linsim*(*l*_1_, *l*_2_) = 1 when *l*_1 _= *l*_2_, and *linsim*(*l*_1_, *l*_2_) = 0, when their minimum subsumer is the root of the ontology. An additional advantage of this measure is that it is bounded between [0, 1]. These fixed bounds are very useful for the incorporation of functional inter-relationships into prediction algorithms, as explained in the proposed approach section.

**Figure 3:**
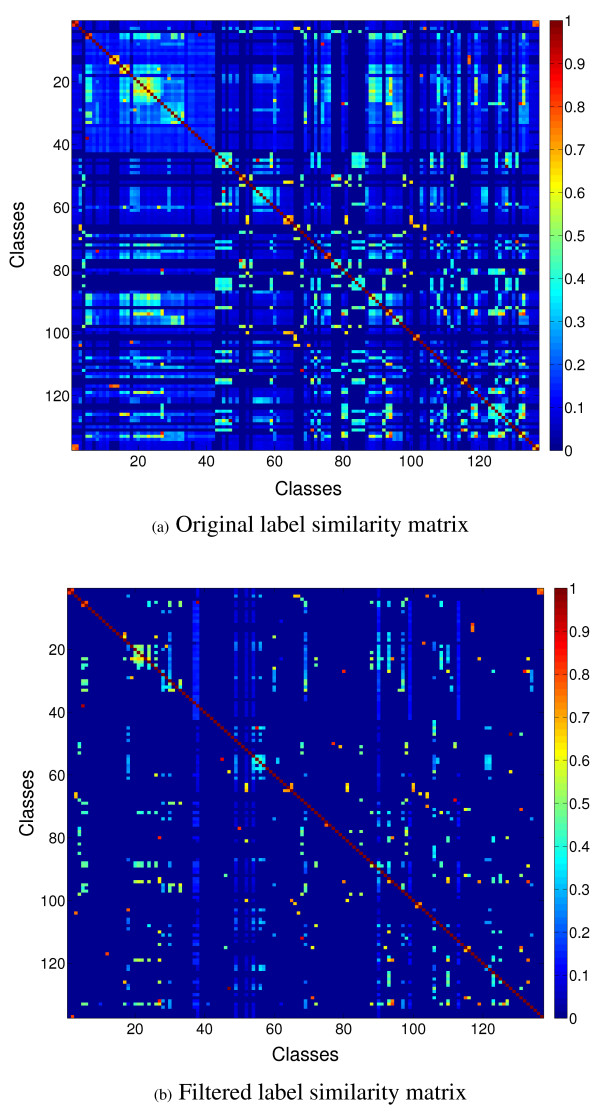
**Original and filtered class/label similarity matrices (labels × labels) generated using the labels of genes in one of the training sets from Mnaimneh *et al *'s data set using Lin's semantic similarity measure** (a) Original label similarity matrix (b) Filtered label similarity matrix.

An example of a label similarity matrix computed for the set of functional classes used in this study is shown in Figure 3(a).

#### k-Nearest Neighbor Classifier

One of the most intuitive classification algorithms is the *k*-nearest neighbor (*k*-NN) classifier [4], which is based on the principle of abundance of the target label in the neighborhood of the query example. We use a weighted variant of this classifier, similar to the direct *k*-NN classifier used by Kuramochi and Karypis [6], which counts the abundance of each label in the neighborhood of size *k *of a protein, weighted by the feature similarity of the neighboring proteins having the corresponding label. Thus, if the feature set of a protein *p *is denoted by *feature*(*p*), then the likelihood score of a label *l *for a protein *p *is given by Equation 2.

(2)

Here *sim*(*feature*(*p*), *feature*(*p*')) denotes the similarity between the feature vectors describing proteins *p *and *p*', and *I *is an indicator function that returns 1 if *l *belongs to the set of labels *p*' is annotated with, 0 for the other labels. Applying this formula for *p *for each label, and then repeating the calculation for all the proteins, produces a |*proteins*| × |*labels*| matrix, named *LL*_*basic*_, of likelihood scores. The performance of this algorithm for each label can then be evaluated using any threshold-free evaluation measure, which was chosen to be the area under the ROC curve (AUC score) [4] in our study.

We chose *k*-NN as the base classifier in our study since it is much simpler than other classification methods, such as SVM [4], and hence it is easier to incorporate additional factors into the model. Also, for the problem of protein function prediction, some authors have reported that with suitable parameter settings, *k*-NN produces comparable performance to SVM [6,26,27].

### Proposed Approach

#### Modified classification algorithm

It can be observed from Equation 2 that *k*-NN is an additive model, *i.e.*, the likelihood scores are obtained by adding the contributions of all the examples in the neighborhood of the test example. Thus, it is intuitively easy to incorporate contributions from examples annotated with *similar *labels. This is implemented in our approach using Equation 3.

(3)

Equation 3 represents a direct extension of the model described in Equation 2, where, in addition to the label being tested (*l*), contributions are also taken from labels similar to *l*. The latter factor is incorporated into the model using the second term , which denotes the sum of Lin's similarities between the target label *l *and all the other labels *l*' assigned to a neighboring protein *p*'. In fact, if *LL*_*basic *_represents the |*proteins*| × |*labels*| like-lihood matrix derived using the direct *k*-NN model (Equation 2), and *LinSim *is the matrix of pairwise label similarities computed using Lin's similarity measure (Equation 1), then the above equation can be written conveniently as follows, where *LL*_*labelsim *_contains the final likelihood scores.

(4)

Equation 4 makes the implementation of our approach much easier.

#### Filtering of label similarities

The label similarity matrix contains a value (however small or large) for each pair of labels. Many of these similarities, especially the smaller ones, are likely to be uninformative, since all the labels (functional classes here) are not expected to interact with all the others, particularly in a large diverse set of labels. Indeed, we observed a significant deterioration in the performance of the label similarity-incorporated classifiers when the raw label similarity matrix is used for incorporating inter-class relationships. In order to handle this issue, we used the following heuristic approach for filtering or sparsifying the label similarity matrix. For each label, we determined a filtering threshold using a cross-validation procedure. This threshold was determined by running a grid search over the interval [0, 1] in steps of 0.05. For each such threshold *t*, all the label similarities for this label that are less than *t *are converted to 0, and a leave-one-out cross-validation procedure is run over the training set to determine the AUC score of the resulting label similarity-incorporated classifier for this label. The threshold that results in the highest AUC score for the resultant label similarity-incorporated classifier is chosen as the filtering threshold for this label. Repeating this process for each label produces a set of thresholds, which is used to generate a filtered version of the original label similarity matrix. A filtered version of the label similarity matrix shown in Figure 3(a) is shown in Figure 3(b).

Note that such a cross-validation-based filtering procedure is expected to produce a more informative version of the original label similarity matrix that is expected to perform well for the unseen test set. However, one issue to consider for this filtering approach is that of consistency between the thresholds computed for the different labels using different training sets. We observed that this issue did not affect our results significantly, but should be considered when applying this approach to other data sets or problems.

## Results and discussion

In this section, we compare the functional relationship-incorporated kNN classifiers with the base kNN classifiers for protein function prediction. This evaluation is conducted using both a cross-validation methodology, as well as a quantitative and qualitative evaluation of the predictions made by these classifiers for independent sets of test proteins. However, before presenting these results, we detail the data sets and the experimental methodology used for these evaluations.

### Data Sets

We used several high-throughput data sets for evaluating our approach. The first was Mnaimneh *et al*'s gene expression data set [13], which measures the expression of all *S. cerevisiae *(budding yeast) genes under a set of 215 titration experiments. The second was another large scale dataset known as the Rosetta gene expression compendium [15] prepared by subjecting yeast cells to a set of 300 diverse mutations and chemical treatments. Pearson's correlation coefficient, used commonly for measuring the similarity between the expression profiles of two genes [28], was used as the feature similarity function *sim *(Equations 2 and 3) for these data sets. We also evaluated our approach on Krogan *et al*'s recently published data set of 7123 highly reliable physical interactions between proteins in yeast [16]. This data set was represented as an *n *× *n *adjacency matrix *A*, with the *A*(*i*, *j*) cell containing the reliability of the interaction between proteins *i *and *j*, if any. We used the *h – confidence *measure for measuring the similarity between the interaction profiles of two proteins in this matrix, which has been shown in a previous study [29] to handle the noise and incompleteness problems of protein interaction data robustly. Finally, we also considered a combined data set, which was prepared by combining the yeast protein interaction data in the BIOGRID database [30] with the two microarray datasets discussed above. This dataset was constructed by preparing the adjacency matrix for the BIOGRID interaction dataset, and concatenating the rows of this matrix with the gene expression profiles of the constituent genes. Also, any columns in the resultant data matrix that have less than two non-zero values are removed, since they do not contribute to the similarity computation. For this data set, we used the cosine similarity measure, since most of the data set is constituted by sparse interaction data.

In addition to these data sets, the structure of the GO biological process ontology, and the GO annotations for *S. cerevisiae *proteins were obtained from the Gene Ontology website  in February, 2008. At this point, only non-IEA annotations were included in the *S. cerevisiae *annotations. These annotations were processed, and all the terms are assigned proteins annotated to any of their descendants, in order to ensure the hierarchical consistency of the annotations. Next, each of the data sets mentioned above is used to construct classification models for a subset of 138 functional classes from the Biological Process ontology of GO (listed in Additional File 1), that have at least 10 members in the corresponding data set. We chose these classes, since, using expert opinion, Myers *et al *[31] have estimated that the predictions made for these classes are likely to be testable in a wet lab and thus are of interest to biologists. Another important reason for the choice of these classes is that no parent-child relationships exist between these classes, and thus it is difficult to use hierarchical relationship-based approaches for these classes. Also, these classes are spread throughout the ontology, and thus are suitable for illustrating the use of semantic similarity to improve predictions by incorporating information from several distant but related functional classes.

Table 1 shows the resultant number of proteins, features and classes used for each data set, as well as the value of *k *for the *k*-NN classifier used in our evaluation. Note that we limited the genes/proteins considered in each of these data sets to those annotated by at least one of the classes considered for the cross-validation experiments.

**Table 1:** Details of the data sets used for evaluating the label similarity-incorporated classifiers using a cross-validation methodology.

**Dataset**	**# Proteins**	**# Features**	**# Classes**	*k*
Mnaimneh	4062	215	137	20
Rosetta	3980	300	137	20
Krogan	2117	2117	108	5
Combined	3762	4277	136	10

### Experimental Methodology

**Figure 4:**
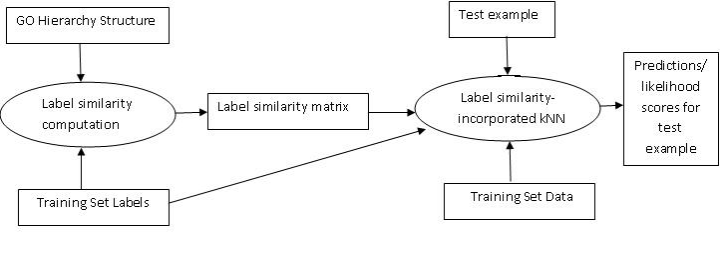
**Overall methodology for predicting the functional annotations of a test protein using the label similarity-incorporated k-NN algorithm**. This methodology is used for our cross-validation experiments, as well as making predictions for test proteins not annotated with any of the target functional classes.

Our overall experimental methodology is shown in Figure 4. Below, we discuss the details of some of the individual components.

#### Computation of label similarity matrix

The first step of our experimental procedure is the construction of the similarity matrix between the labels, or the inter-relationship matrix between the corresponding classes, for each of the above data sets. For each set of labels, the original set of annotations are collected from the yeast GO annotations, and the |*proteins*| × |*labels*| binary matrix corresponding to the functional classes used for each of the data sets is prepared, where a 1 denotes that a protein is annotated with the particular class, and 0 otherwise. Equation 1 is then applied to all pairs of labels in this matrix to obtain the final label similarity matrix. Note that only the training examples are used for computing this matrix, both in the cross-validation and independent test set prediction experiments. An example of the matrix constructed from one of the training sets derived from Mnaimneh *et al*'s data set is shown in Figure 3(a).

#### Classification and evaluation

Here, for each protein *p *in the test set, Equation 3 is used to calculate the likelihood score for *p *to be annotated with each class *c*. In the cross-validation experiments, where a five-fold cross-validation procedure is followed, repeating this process for each protein in each fold, using the other four folds as training sets, produces the global protein-label likelihood score matrix, which can then be evaluated by computing an AUC score for each label. Although the results reported for these experiments are based on five-fold cross validation, we obtained very similar results with other fold configurations also. The use of this methodology for making predictions in the independent test set experiments is straightforward, and is described in detail in a later section. Also, the values of *k *chosen for each data set, shown in the last column of Table 1, is chosen in accordance with the density or the sparsity of the corresponding data set. Thus, *k *is chosen to be high (20) for the dense microarray data sets, low (5) for the sparse protein interaction data set (Krogan), and an intermediate value (10) for the combined dataset constructed by combining both microarray and interaction data. However, we obtained similar results using other values of *k*. Finally, an important intermediate step in our method is the filtering of the original label similarity matrix, which is implemented as explained in the Proposed Approach section earlier.

Using this general classification framework, we evaluated the performance of the base kNN classifiers and the functional or label similarity-incorporated classifiers using two validation methodologies. In the first set of experiments, we used a five-fold cross-validation methodology for this evaluation on the four data sets detailed in Table 1. In the second set, we used these portions of the Rosetta and Mnaimneh data sets as training sets, and made predictions for the proteins covered in the GO annotation database but not in the cross-validation experiments. Using annotations added to the GO database between February–September 2008, we validated these predictions quantitatively, and also examined independent evidence that validated three of these novel predictions biologically. The following two sections discuss the results from these two evaluation methodologies in detail.

### Results from cross-validation experiments

In this section, we compare the performance of the label similarity-incorporated classifier with the base *k*-NN classifier using a five-fold cross-validation approach, and illustrate how the use of inter-relationships between classes can help improve the accuracy of predictions made over a set of target classes. Note that all the AUC scores and the associated statistics presented in this section are obtained as the average of fifty five-fold cross validation runs of each classifier, unless otherwise stated.

#### Improvement of performance for a large set of classes

Table 2 lists specific comparative statistics about the AUC scores obtained for all the classes using the base *k*-NN classifiers and their label similarity-incorporated versions. As can be seen, a non-trivial improvement is observed in the average AUC score over all the classes for all the data sets, and the maximum improvement on at least one of the classes is usually very high. For instance, for the Mnaimneh data set, an average improvement of 3.57% is observed for all the classes, while the maximum improvement in AUC over one of the classes is 0.1882, which accounts for a nearly 40% improvement.

**Table 2:** Statistics about the comparative performance of the base *k*-NN classifiers and their label similarity-incorporated versions, measured in terms of the number of classes for which AUC scores are improved by the latter over the former, and the average and maximum improvement in AUC scores over all classes.

**Dataset**	**Total # classes**	**# Classes improved**	**Average improvement over all classes**	**Maximum improvement**
Mnaimneh	137	74	0.0219 (3.57%)	0.1882 (39.92%)
Rosetta	137	47	0.0083 (1.33%)	0.2091 (38.66%)
Krogan	108	30	0.0045 (0.63%)	0.1982 (31.82%)
Combined	136	59	0.0079 (1.02%)	0.1129 (20.39%)

**Figure 5:**
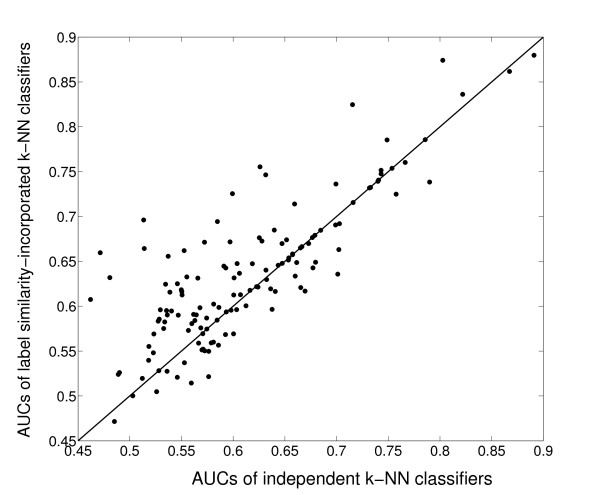
**Comparison of the performance of functional similarity-incorporated *k*-NN classifiers with individual *k*-NN classifiers for Mnaimneh *et al *'s data set**. The X-axis shows the AUC scores of the base classifiers for each class, and the Y-axis denotes the AUC scores of their label similarity-incorporated classifiers.

We also examined the effect of our approach on the performance of classification for each class individually. Figure 5 shows the comparison of performance of individual base *k*-NN classifiers for each functional class, and their functional similarity-incorporated versions for Mnaimneh *et al*'s data set. In this figure, the AUCs of individual *k*-NN classifiers for each class are plotted on the x-axis, while those of the functional similarity-incorporated *k*-NNs are plotted on the y-axis. Thus, the points above the *y *= *x *line indicate an improvement in the AUC score of the corresponding class, and vice versa. Using this interpretation, it is easy to see from this plot that the performance of a substantial fraction of the classes (74/137) are improved by incorporating contributions from similar classes. Another encouraging aspect of this plot is that almost none of the classes suffers a major loss of prediction accuracy due to the incorporation of label-similarity, and in most cases, the difference can be accounted for by the effect of randomization in the cross-validation process. This implies that for those classes whose performance is invariant, the label similarity filtering process is able to infer that incorporating label similarity is not appropriate for these classes, and does not identify irrelevant relationships in the filtered label similarity matrix. Similar results are obtained for the other data sets as well.

#### Improvement of performance for small classes

One of the primary motivations for the incorporation of label similarity into standard function prediction algorithms was to improve the prediction accuracy for data-poor classes, as discussed in the Background section earlier. Our approach is expected to be useful for this task, as, in our model, the small classes can seek a contribution from classes of bigger sizes that have a high semantic similarity with them. To test this hypothesis, we selected classes of size at most 30 in all the data sets being used, and analyzed the results obtained using label similarity, as against those from basic classification. Table 3 provides detailed statistics about these results. Indeed, it can be seen from these results that the improvements, both in absolute terms and as a percentage of the average AUC score of the base classifiers, for these classes are significantly higher than the corresponding figures in Table 2. This shows that the label similarity-based classification approach is indeed able to help improve the accuracy of the predictions made over data-poor classes, for which it is hard to build very accurate base classifiers.

**Table 3:** Statistics about the comparative performance of the base *k*-NN classifiers and their label similarity-incorporated versions on small classes (size ≤ 30), measured in terms of the number of classes for which AUC scores are improved by the latter over the former, and the average and maximum improvement in AUC scores over all classes.

**Dataset**	**# Small classes**	**# Classes improved**	**Average improvement over all small classes**	**Maximum improvement**
Mnaimneh	47	27	0.0358 (6.24%)	0.1882 (39.92%)
Rosetta	48	21	0.0225 (3.82%)	0.2091 (38.66%)
Krogan	40	14	0.0129 (1.89%)	0.1982 (31.82%)
Combined	48	28	0.0197 (2.72%)	0.1129 (20.39%)

**Figure 6:**
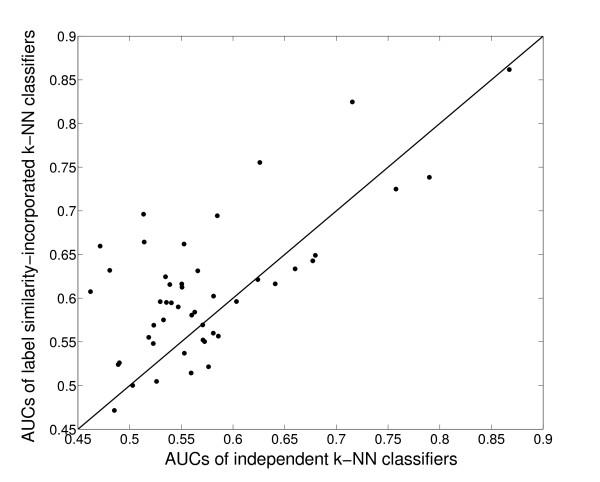
**Comparison of the performance of functional similarity-incorporated *k*-NN classifiers with individual *k*-NN classifiers for small classes (size ≤ 30) in Mnaimneh *et al *'s data set**. The X-axis shows the AUC scores of the base classifiers for each class, and the Y-axis denotes the AUC scores of their label similarity-incorporated classifiers.

The class-by-class improvements for each small class in Mnaimneh *et al*'s data set are shown graphically in Figure 6. While the performance of some classes is invariant (close to the *y *= *x *line), several classes show a large improvement in performance. In particular, we investigated the class *GO:0051049 *(*regulation of transport*), which has only 11 members in Mnaimneh *et al*'s data set, and shows the maximum AUC improvement of almost 40%. In order to identify the classes that contributed to the improved performance of this class (besides itself), we identified the classes that had a non-zero semantic similarity with this class in the filtered label similarity matrix shown in Figure 3(b). Table 4 provides details of the eight classes so found.

**Table 4:** Details of the classes most similar to GO:0051049 (*regulation of transport*) that are found to help improve the prediction accuracy of this class in Mnaimneh *et al*'s data set.

**GO Term**	**Definition**	**Size**	**Similarity with target class**
GO:0016192	Vesicle-mediated transport	328	0.4085
GO:0016458	Gene silencing	100	0.4067
GO:0016481	Negative regulation of transcription	163	0.4317
GO:0040029	Regulation of gene expression	100	0.4067
GO:0045184	Establishment of protein localization	273	0.4014
GO:0045941	Positive regulation of transcription	102	0.4143
GO:0051052	Regulation of DNA metabolic process	80	0.4058
GO:0051252	Regulation of RNA metabolism	383	0.4743

As can be observed from Table 4, all the classes contributing to the improvement of predictions made for GO:0051049 are fairly large in size, and their high semantic similarity with the target class enables the label similarity-incorporated classifier to make use of the members of these classes to acquire more information about the data-poor class. Also, interestingly, most of these classes are biologically related to the target class, since most of them are related to the processes of *transport *(*vesicle-mediated transport *and *establishment of protein localization*) and *regulation *(*regulation of DNA metabolic process*, *regulation of RNA metabolism *etc). These semantic relationships are shown graphically in Additional File 2, which shows the positions of these classes in the biological process ontology, and their structural relationships in the ontology suggest that it is useful to incorporate such relationships into the prediction process for small classes, such as the one discussed in this example.

This analysis also supports our hypothesis that the label similarity filtering process is able to capture the most meaningful relationships between functional classes in a given label similarity matrix, and the label similarity-incorporated classification process is able to utilize these relationships to improve the predictions over individual classes.

#### Incorporating information in the whole GO biological process ontology

Unlike the hierarchical consistency enforcement approach discussed in the related work section, which focuses on the subgraph of the functional hierarchy corresponding to the target classes, one of the advantages of the direct incorporation of relationships into the classification model is that relationships in the entire hierarchy can be incorporated into the classification model, while holding the set of target classes constant. This can be done by simply modifying the label similarity matrix to include the semantic similarities between the target classes and all the other classes in the hierarchy. Thus, instead of using an |*l*| × |*l*| matrix of similarities, one can use a |*l*| × |*L*| matrix, where |*l*| is the number of target classes, and |*L*| is the number of all the (non-empty) classes in the hierarchy. The rest of the approach, as shown in Figure 4, remains the same.

We tested this idea for Mnaimneh *et al*'s data set, using the GO biological process ontology as the source of all the functional inter-relationships, which produced a 137 × 2395 label similarity. The results of this experiment, generated using ten rounds of five-fold cross validation, are summarized in Table 5, for all the classes and for the small classes. It can be observed that these results are comparable to those obtained from Mnaimneh *et al*'s data set using only the target classes for identifying functional relationships. However, some results are improved when the whole hierarchy is used, namely the average and the maximum improvement over the small classes (6.32%-vs-6.24% and 53.93%-vs-39.92% respectively), showing once more that the small classes are able to utilize the label similarity matrix more effectively. However, it is important to carefully identify the relationships to be utilized due to the very large number (137 × 2395) of possible relationships, many of which are expected to be uninformative. This task may need a more sophisticated methodology than that used for only the target classes which had fewer (137 × 137) possible relationships.

**Table 5:** Statistics about the comparative performance of the base *k*-NN classifiers and their label similarity-incorporated versions using information in the whole GO biological process ontology for all as well as small (size ≤ 30) target classes in Mnaimneh *et al*'s data set, measured in terms of the number of classes for which AUC scores are improved by the latter over the former, and the average and maximum improvement in AUC scores over all classes.

	**Total # classes**	**# Classes improved**	**Average improvement over all classes**	**Maximum improvement**
All classes	137	71	0.0167 (2.65%)	0.2492 (53.93%)
Small classes	47	29	0.0363 (6.32%)	0.2492 (53.93%)

In summary, these cross-validation-based results show that the incorporation of direct relationships between functional classes constituting the GO functional hierarchies, measured using a suitable semantic similarity measure, is a useful method for improving the accuracy of the predictions made over a set of target classes, particularly for classes with a small number of member proteins.

#### Comparison with Yu *et al*'s GEST approach

As mentioned before, Yu *et al *proposed the GEST approach [22] for incorporating semantic similarities between functional classes into a protein function prediction algorithm. They used two different semantic similarity measures, namely PK-TS and SB-TS, in this study. Here, we present a comparison of the results produced by our label similarity-incorporated classification algorithm and GEST for our target set of 138 classes.

In the first experiment, we compared the accuracy of the predictions of our approach and GEST in the case where only the similarities between the target classes are incorporated into the prediction algorithm, corresponding to the results presented in the *Improvement of performance for a large set of classes *section. Note that the SB-TS measured used in GEST is not applicable in this experiment, since it only considers similarities between classes that have an ancestor-descendant relationship between them, and our set of target classes do not have any such relationships among them. Thus, the results presented here are generated using the PK-TS similarity measure. Table 6 provides comparative statistics about the AUC scores obtained for all the target classes using our approach and GEST on all the test data sets with the same parameter values as used earlier.

**Table 6:** Statistics about the comparative performance of GEST and our label similarity-incorporated kNN classifiers, measured in terms of the number of classes for which AUC scores are improved by the latter over the former, and the average and maximum improvement in AUC scores.

**Dataset**	**Total # classes**	**# Classes improved**	**Average improvement over all classes**	**Maximum improvement**
Mnaimneh	137	87	0.0116 (1.86%)	0.1788 (37.83%)
Rosetta	137	65	0.0004 (0.06%)	0.1854 (36.6%)
Krogan	108	60	0.0059 (0.84%)	0.1307 (23.53%)
Combined	136	75	0.0081 (1.04%)	0.2117 (48.85%)

The statistics in Table 6 show that for a substantial number of classes, more than half in most cases, our approach is able to produce more accurate predictions than GEST, particularly in terms of the maximum improvement over at least one of the classes. This overall improvement is illustrated in greater detail in Additional File 3, where the AUC scores for each class considered for Mnaimneh *et al*'s data set using our method is plotted against those generated by GEST. This plot shows that several classes indeed obtain a significantly high improvement in the AUC score using our method.

In another set of experiments, we compared the accuracy of the predictions made by our method and GEST, using the similarity between the target functional classes for Mnaimneh *et al*'s data set and all the non-empty GO Biological Process classes, using a methodology similar to the one used in the *Incorporating information in the whole GO biological process ontology *section. The SB-TS measure was also used in these experiments, since all the relationships in the Biological Process ontology are considered here. Table 7 details the results of the comparison between the results produced by our method and those by GEST-PK-TS and GEST-SB-TS.

**Table 7:** Statistics about the comparative performance of GEST classifiers and our label similarity-incorporated classifiers using information in the whole GO biological process ontology, measured in terms of the number of classes for which AUC scores are improved by the latter over the former, and the average and maximum improvement in AUC scores.

**Method**	**Total # classes**	**# Classes improved**	**Average improvement over all classes**	**Maximum improvement**
GEST-PK-TS	137	75	0.0235 (3.82%)	0.2171 (42.04%)
GEST-SB-TS	137	77	0.0144 (2.34%)	0.2137 (47.4%)

Similar to the results discussed earlier, the results in Table 7 also show that our method is able to improve the results of GEST for a significant number of classes. These improvements in both the set of experiments can be explained on the basis of the following two differences between our and GEST's methodologies:

• The semantic similarity measures used in GEST are only computed on the basis of the structure of the ontology, while the formulation of Lin's similarity measure used in our method also takes the set of members at each node into account. This can help uncover similarities between nodes that are otherwise reasonably distant in the ontology, but have very similar set of proteins contained in them, thus indicating a semantic relationship between them that is not captured by the structure of the ontology.

• GEST uses the maximum of the similarities of all the annotations of a neighborhood protein to the target class as the contribution to the score of the target protein for this class. On the other hand, our cross validation-based method for filtering the label similarity matrix is more adaptive, since it determines which classes should contribute to the score of a given target class on the fly. This can help avoid collecting spurious contributions due to small values of similarity between the target and the contributing classes.

These conceptual differences lead to the differences between the results produced by our method and Yu *et al*'s GEST method. However, we would like to stress here that for several classes, GEST is able to produce more accurate predictions than our method. This indicates the possibility for the development of a hybrid system that makes the best use of both the methods, although this is beyond the scope of the current study.

### Validation of function predictions for proteins in the test set

One of the important distinctions between our approach and some other approaches that address the problem of incorporating functional inter-relationships into function prediction algorithms [20,21] is that we perform this incorporation into classifiers that make predictions based on genomic data instead of annotation patterns. This offers the important advantage of the ability to make predictions for proteins that have not been assigned any functional annotations so far. In order to illustrate this advantage, we tested if the ability of the label similarity-incorporated classifiers to make more accurate predictions than the base classifiers, demonstrated in the previous section through cross-validation experiments, can help uncover the annotations of currently unannotated proteins. We tested this hypothesis both quantitatively, using a historical rollback-type strategy [21], and qualitatively, by showing that three predictions made by the label similarity-incorporated are supported by independent evidence from the literature.

In the quantitative evaluation, we made predictions for proteins not used in the cross-validation experiments, i.e., those proteins that were not annotated with any of the classes considered, using the set of proteins considered in cross-validation as the training set. In order to use our approach for this task, we randomly split the original training set in an 80 – 20 ratio, with the larger set serving as the final training set. The smaller set is used as a validation set for determining the thresholds to be used for filtering the label similarity matrix. The likelihood scores generated for the test set using both the base kNN and the label similarity-incorporated kNN classifiers for the classes considered were averaged over ten rounds. This computation was performed for the Mnaimneh and Rosetta gene expression data sets to maximize the coverage of the test set, and the same value of *k *= 20 as for the cross-validation experiments, was used here. Also, we used only the version of the label similarity matrix containing pairwise similarities between the classes over which predictions are being made, such as the one shown in Figure 3(a).

The scores produced by both the classification algorithms (base and label similarity-incorporated) were ranked in descending order independently for each class, and proteins with the same score (mostly in the case when the score is 0) were sorted by their ORF name. The complete ranked lists of predictions for the Mnaimneh and Rosetta data sets are available as Additional Files 4 and 5 respectively. Now, since we had used the current annotations as of February, 2008 in constructing the training set, we used the annotations added for these classes to the GO database between February and September, 2008 to evaluate the accuracy of these predictions. More specifically, we compared how the ranks of accurate predictions compared between the base kNN and the label similarity-incorporated classifiers. Table 8 details the results obtained from this validation. As can be noted, the median ranks assigned to the valid predictions by the label similarity-incorporated classifiers are significantly lower than that by the base kNN classifiers, showing that an accurate prediction is uncovered much earlier using label similarity than by the base classifiers. The difference between the distribution of the entire set of ranks of validated predictions is quantified by a p-value, which is computed using Wilcoxon's signed-rank test for comparing two paired distributions [32]. These p-values are also low, particularly for Mnaimneh's data set (0.0047), indicating that these sets of ranks are significantly different. Combining these pieces of evidence, it can be said that label similarity-incorporated classifiers generally assign better ranks to annotations that are subsequently found to be valid.

**Table 8:** Statistics about the predictions made by the base kNN classifiers and their label similarity-incorporated versions for the genes in Mnainmeh and Rosetta data sets not covered in the cross-validation experiments, and how many of these predictions could be validated based on the annotations added to the GO database between February–September 2008.

**Dataset**	**# Test genes**	#**# Validated predictions**	**Median of ranks in label similarity kNN**	**Median of ranks in base kNN**	**P-value**
Mnaimneh	1621	1003	497	637	0.0047
Rosetta	1609	998	509	560	0.319

**Figure 7:**
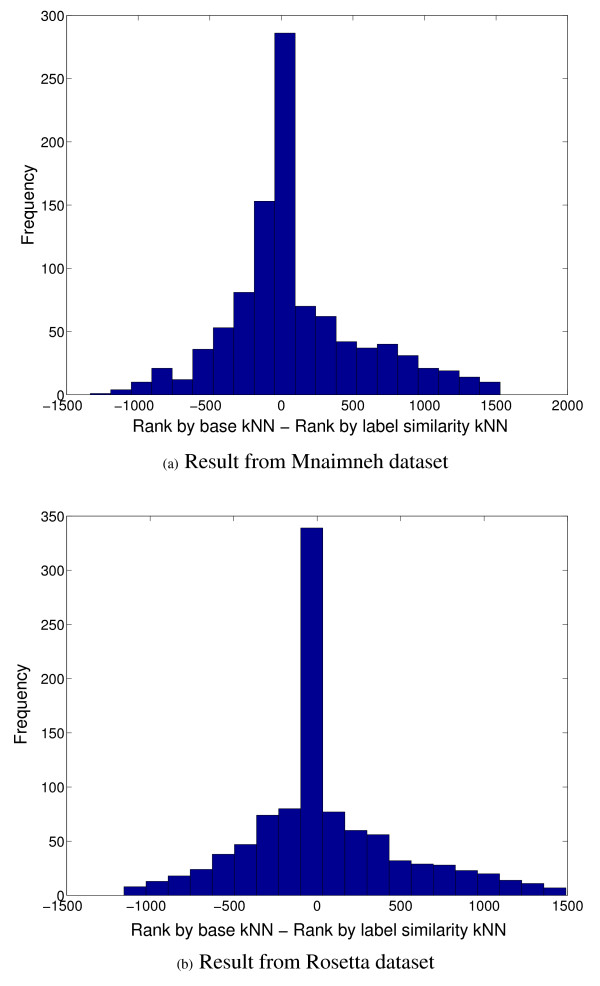
**Histograms showing the distribution of differences between the ranks assigned by the base kNN and label similarity-incorporated classifiers to functional annotations for a set of test proteins [(rank by base classifier)-(rank by label similarity-incorporated classifier)] that have been validated in the GO database between February–September 2008 for the Mnaimneh and Rosetta data sets **(a) Result from Mnaimneh dataset (b) Result from Rosetta dataset.

Looking further into these results, we examined the distribution of the difference between the ranks assigned by the base and label similarity-incorporated classifiers to the valid annotations, i.e. (rank by base kNN)-(rank by label similarity-incorporated kNN), shown in Figures 7(a) and 7(b) for the Mnaimneh and Rosetta data sets respectively. It can be seen from these plots that in the cases where the label similarity-incorporated classifiers assign lower ranks than those assigned by the base classifiers (rank difference > 0), the difference is significantly higher than that in the converse cases (rank difference < 0). In numbers, the median difference of ranks in the former case for Mnaimneh's data set is 345, while that in the converse case in 151, and the numbers for the Rosetta data set for these cases are 308 and 190 respectively. This shows that for the valid predictions, whenever the label similarity-incorporated classifiers assign a worse rank than the base classifiers, the difference is significantly smaller than in the converse case.

**Figure 8:**
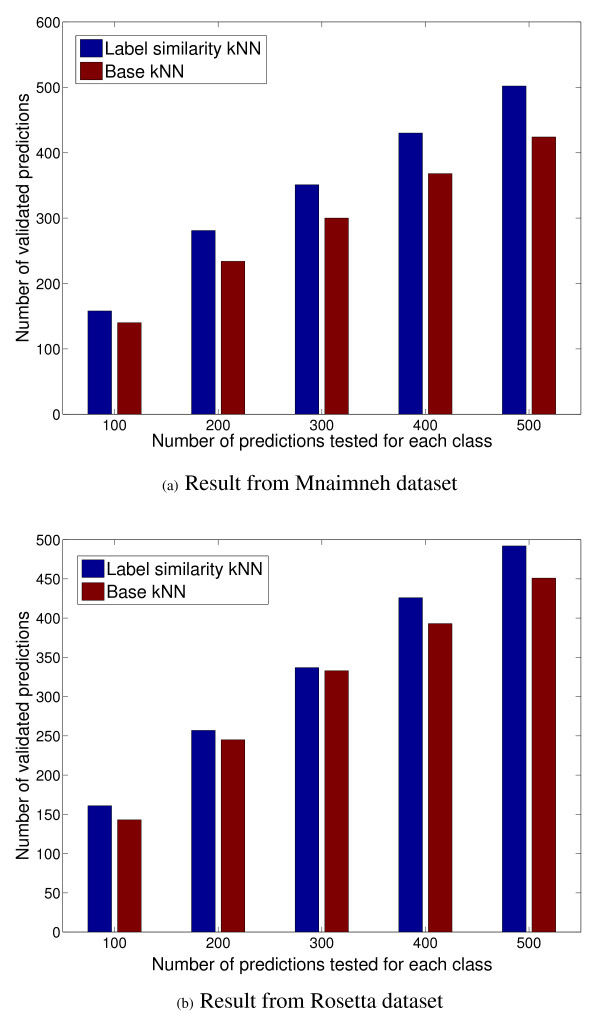
**The number of predictions validated by the GO database between February–September 2008 among the top *n *ranks assigned by the label similarity-incorporated and base kNN classifiers of each target class for the Mnaimneh and Rosetta data sets **(a) Result from Mnaimneh dataset (b) Result from Rosetta dataset (best seen in color).

As the final test in the historical rollback-based evaluation, we examined how many of the top-*n *ranked predictions (*n *= 100, 200,..., 500) for each class could be validated using the annotations added to GO between February–September 2008. Figures 8(a) and 8(b) show these numbers of accurate predictions made from the Mnaimneh and Rosetta data sets respectively. It can be seen clearly that for every value of *n*, the label similarity-incorporated classifiers are able to make more accurate predictions than the base classifier. This again shows that the incorporation of functional inter-relationships into function prediction algorithms can help improve both the precision and recall of valid functional annotations, even for proteins currently not members of the functional classes considered.

These results quantitatively illustrate the effectiveness of incorporating label similarity into protein function prediction algorithms for uncovering the functions of unannotated proteins. To show that this incorporation can also help characterize some proteins for which little functional information is available, we investigated the predicted functions for several uncharacterized proteins to determine whether there is independent evidence to support our predictions. Indeed, we found several instances where predictions unique to the label similarity-incorporated classifiers are supported by compelling independent evidence, which makes these proteins promising candidates for further experimental validation. These examples are discussed in detail below.

#### Possible novel link between vacuolar [H+]-ATPase dissociation and microtubules supported by YDL123W

YDL123W (Sna4) is a protein that is known to localize to the outer vacuolar membrane, but its biological role has not yet been characterized [33]. Our label similarity-based classifier, based on the Rosetta expression dataset, made a high-confidence prediction of this protein to be involved in the function GO:0031023 (*microtubule organizing center organization and biogenesis*), ranking it as the 3rd highest prediction. This prediction is interesting, since the vacuolar ATPase is known to dissociate in yeast in response to glucose depletion [34]. This process of dissociation has not been extensively characterized, but recent studies have demonstrated that nocodazole, a drug that disrupts microtubules, specifically inhibits v-ATPase dissociation [34]. Thus, we hypothesized that Sna4 may support the microtubule-based dissociation of the v-ATPase. This hypothesis is supported by independent high-throughput evidence. For example, Sna4 shares physical interactions with three components of the V1 domain of the v-ATPase (Vma13, Vma7, Vma8) [35]. In addition, Sna4 interacts physically with Hxt1 [35], a low-affinity glucose transporter, which may suggest a connection to the main environmental signal known to signal v-ATPase dissociation, i.e., glucose deficiency. We were unable to find any specific Sna4 connections to microtubule-related proteins, but the fact that this prediction appeared in the high-confidence region of this particular GO term along with independent physical evidence supporting a potential role in v-ATPase dissociation suggest that this is a promising candidate for further experimental exploration and validation. Notably, this prediction was only made by the label similarity-incorporated classifier, and it appeared at a low rank of 403 in the predictions from the base kNN classifier for this term. The AUC score for this term, to which only 16 members in the Rosetta data set were known to be associated, was 0.5949 using the label similarity-incorporated classifier, as compared to 0.4604 using its base kNN classifier.

#### YDR233C may play a previously uncharacterized role in mannoprotein metabolism

Glycoproteins are an important class of proteins characterized by the addition of oligosaccharide chains to their polypeptide side-chains. Glycosylation of these proteins typically plays an important role in their function, and they are known to be central for several cellular functions including the recognition of cell structures, inter-cell signaling and transport [36]. Mannoproteins are glycoproteins with high enrichment for mannose oligosaccharides, and in yeast, these have been specifically associated with regulation of the porosity of the cell wall [37].

Our label similarity-incorporated classifier predicted the previously uncharacterized protein YDR233C (Rtn1) to have a strong association to the term GO:0006056 (*mannoprotein metabolism*) based on the Rosetta expression dataset. Rtn1 has been localized to the endoplasmic reticulum (ER) and ER membrane in yeast [38], but little else is known about its function, as indicated by no annotations in either the molecular function or biological process ontologies in GO. We found its predicted role in mannoprotein metabolism interesting because glycoprotein biosynthesis is largely carried out by the ER, and specifically relies on the activity of proteins in the ER membrane [36,39,40], which is consistent with the confirmed localization of Rtn1. Another important piece of independent evidence for our prediction of Rtn1 involvement in mannoprotein metabolism is its reported genetic interaction (phenotypic enhancement) with Dpm1 [41], an essential protein known to be involved in mannose-specific glycosylation in the ER [36]. Due to these pieces of strong evidence, we suspect that Rtn1 would be a promising direction for follow-up experiments for biologists interested in mannoprotein metabolism. Again, this prediction is unique to the label similarity-incorporated classifier for this term, as it appeared as the 392nd highest prediction by the base kNN classifier but the 9th highest prediction by the label similarity-incorporated classifier. The AUC score for this term, to which only 11 members in the Rosetta data set were known to be associated, was 0.7136 using the label similarity-incorporated classifier, as compared to 0.5366 using its base kNN classifier.

#### Elucidation of an uncharacterized protein's involvement in RNA processing

YHR156C (Lin1) is a protein whose function is largely uncharacterized (no current biological process or molecular function annotations in GO). Interestingly, Lin1 appeared at the top of several lists of predictions made by the label similarity-incorporated classifiers for processes related to RNA metabolism or processing, including snoRNA metabolism (GO:0016074), RNA 3'-end processing (GO:0031123), and mRNA metabolism (GO:0016071). The same protein was not predicted at a similar confidence level by the base classifiers for these classes (792nd, 865th and 17th respectively).

Interestingly, although this protein's function is not captured by current biological process or molecular function annotations, it is known to be involved in the U5 small ribonucleoprotein (snRNP), which is a component of the spliceosome in yeast [42]. The spliceosome is a highly conserved nuclear component involved in pre-mRNA splicing [42,43]. Given Lin1's involvement in the spliceosome, its association with general mRNA metabolism is not surprising or particularly novel, but it does provide good validation of our methodology of incorporating functional inter-relationships into function prediction algorithms. However, we do find it interesting that Lin1 is predicted more specifically to play a role in snoRNA metabolism and RNA 3'-end processing. snoRNAs are typically encoded within introns of other genes, and recent evidence has shown a striking dependence of splicing efficiency on the proximity of the 3'-end of the snoRNA and the intron branch point [44]. Thus, our predictions might suggest a more specific role of the U5 snRNP in the production of snoRNAs.

All of these validations represent instances where a protein with relatively shallow previous characterization has been associated with a specific GO term using our prediction methodology, which has allowed us to generate non-trivial hypotheses about its cellular role. Furthermore, all of the functional associations discussed here were unique to the label similarity-incorporated classifiers, which indicates that such an approach can be used to predict reliable, specific, and novel biology. We have provided the ranked prediction lists for both the versions of the classifiers as Additional Files 4 and 5, which may help in the discovery of novel functional annotations other than those discussed above.

In summary, the cross-validation experiments and the quantitative and qualitative evaluation of the predictions for previously unannotated proteins shows how the incorporation of inter-relationships between functional classes into standard function prediction algorithms can help expand the set of annotated proteins in *S. cerevisiae *and other genomes to include proteins for which currently no or very little functional information or annotations are available.

## Conclusion

In this paper, we demonstrated the utility of incorporating functional interrelationships into protein function prediction algorithms, in order to improve the predictions made by them. We modeled these relationships using Lin's semantic similarity measure [14] and modified the commonly used k-nearest neighbor classification algorithm in order to seek contributions from other classes, weighted by their semantic similarity with the target class. Cross-validation results on several large genomic data sets showed that this approach is able to improve the results for a large majority of the classes considered. In particular, a bigger improvement was seen for smaller classes, which are otherwise harder to model and predict. In addition, we also provided qualitative and quantitative evidence that this incorporation of functional inter-relationships enables the discovery of interesting biology in the form of novel functional annotations for several yeast proteins, such as Sna4, Rtn1 and Lin1.

Our work can be extended in several directions. It will be useful to incorporate the concept of functional similarity into SVMs, which do not have the additive characteristic like *k*-nearest neighbor, and other function prediction algorithms, such as FunctionalFlow [45] for protein interaction networks. Another important direction will be to carefully analyze the relationships between a set of target classes with all the other classes in the hierarchy, in order to incorporate more information into the classifiers, while reducing the effect of spurious relationships. As noted in the related work section, incorporating both parent-child and more distant relationships between classes into function prediction algorithms will be required for making optimal use of relationships constituting GO. For this, it will be useful to integrate our framework with the Bayesian network-based approach of Barutcuoglu *et al *[17] for enforcing parent-child consistency between the results of standard prediction algorithms. As an example of a possible methodology of integrating these approaches, distant functional relationships could be incorporated first using our technique, and then the resulting likelihood scores could be propagated hierarchically using the Bayesian network approach. Investigation of such schemes will be a topic of our future research.

## Availability and requirements

**• Project Home Page**: 

**• Operating System(s)**: Platform independent.

**• Programming language**: Matlab (Tested for version 7.4 and above, but expected to work with earlier versions also).

**• License**: None.

**• Any restrictions to use by non-academics**: This paper must be cited.

## Authors' contributions

GP, CLM and VK conceived the study and developed the proposed approach and the evaluation methodologies. GP prepared the implementation and experimental results. CLM helped in interpretation and literature validation of the predictions. All the authors participated in the preparation of the manuscript and approved the final version.

## Supplementary Material

Additional file 1 **Details of the GO classes used for evaluation**. Details of the 138 functional classes from the GO Biological Process ontology whose subsets (classes having at least 10 members in the corresponding data set) are used for evaluation using several genomic data sets in this study. Click here for file

Additional file 2 **Arrangement of the functional classes aiding the improvement of the AUC score of the GO:0051049 (regulation of transport) class in the GO biological process ontology**. This figure shows the arrangement of the functional classes aiding the improvement of the AUC score of the GO:0051049 (regulation of transport) class (listed in Table 4) in the GO biological process ontology. Their structural proximity to the target class (GO:0051049) suggests their potential to help improve the predictions for this class. Click here for file

Additional file 3 **Comparison of AUC scores from our approach and GEST**. This figure shows the comparison of the performance of our functional similarity-incorporated *k*-NN classifiers with individual GEST classifiers for Mnaimneh *et al*'s data set. Click here for file

Additional file 4 **Ranked list of predictions from the Mnaimneh gene expression data set**. A detailed list of ranked predictions produced by the label similarity-incorporated kNN classifiers (first worksheet) and base kNN classifiers (second worksheet) for the test genes extracted from the Mnaimneh gene expression data set. The GO terms, arranged in columns, are sorted from left to right in the order of decreasing AUC improvements by incorporating functional relationships into their base classifiers. The genes in each column are ranked in descending order by the score assigned by the corresponding kNN classifier. Genes with the same score (mostly in the case when the score is 0) are sorted by their ORF name. Click here for file

Additional file 5 **Ranked list of predictions from the Rosetta gene expression data set**. A detailed list of ranked predictions produced by the label similarity-incorporated kNN classifiers (first worksheet) and base kNN classifiers (second worksheet) for the test genes extracted from the Rosetta gene expression data set. The GO terms, arranged in columns, are sorted from left to right in the order of decreasing AUC improvements by incorporating functional relationships into their base classifiers. The genes in each column are ranked in descending order by the score assigned by the corresponding kNN classifier. Genes with the same score (mostly in the case when the score is 0) are sorted by their ORF name. Click here for file
