# Supplementary figures and images for: Incorporating functional inter-relationships into protein function prediction algorithms
Source: BMC Bioinformatics. 2009 May 12;10:142. doi: 10.1186/1471-2105-10-142 (PMC2693438; doi:10.1186/1471-2105-10-142)

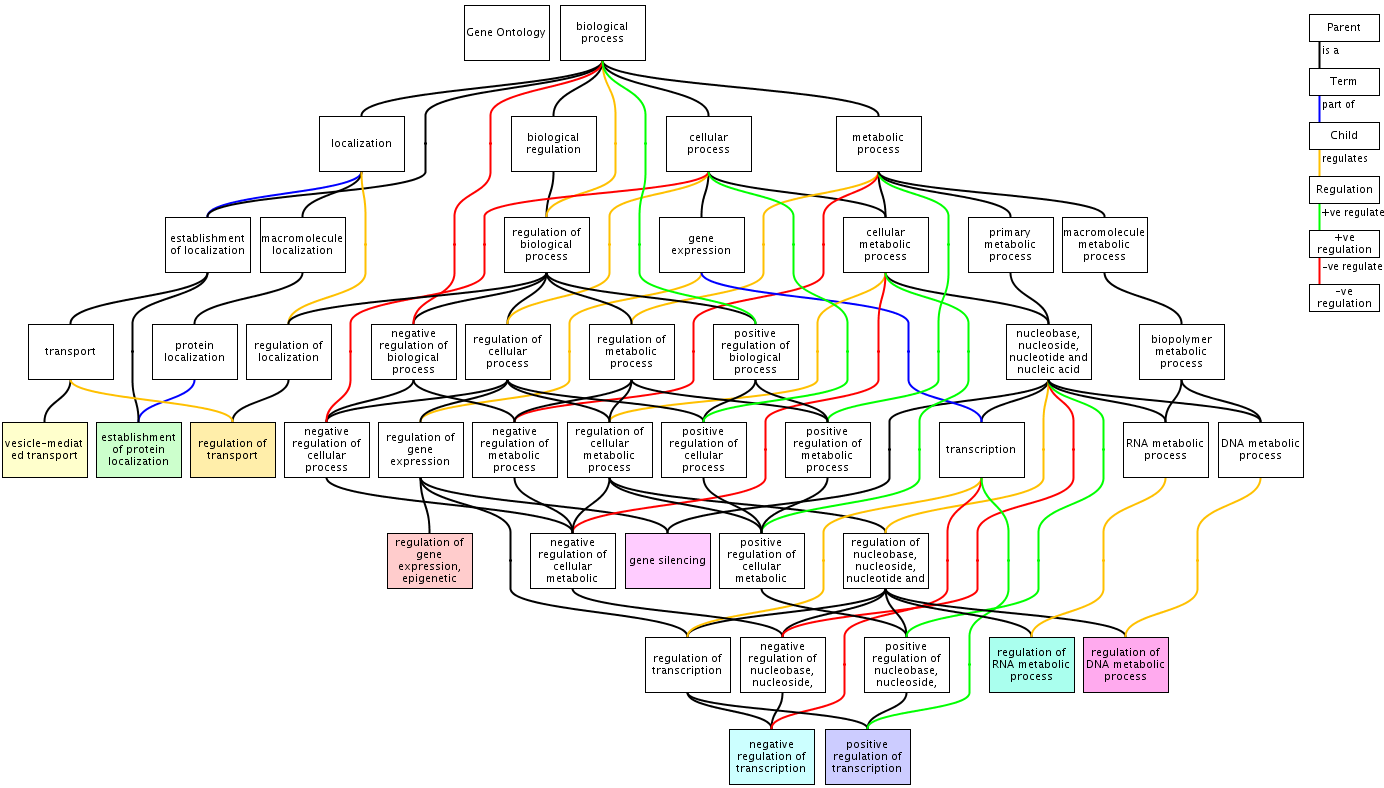

Supplement: Additional file 2 — Arrangement of the functional classes aiding the improvement of the AUC score of the GO:0051049 (regulation of transport) class in the GO biological process ontology. This figure shows the arrangement of the functional classes aiding the improvement of the AUC score of the GO:0051049 (regulation of transport) class (listed in Table 4) in the GO biological process ontology. Their structural proximity to the target class (GO:0051049) suggests their potential to help improve the predictions for this class. [file 1471-2105-10-142-S2.png]
